# Correlation of objective image quality metrics with radiologists’ diagnostic confidence depends on the clinical task performed

**DOI:** 10.1117/1.JMI.12.5.051803

**Published:** 2025-04-11

**Authors:** Michelle C. Pryde, James Rioux, Adela Elena Cora, David Volders, Matthias H. Schmidt, Mohammed Abdolell, Chris Bowen, Steven D. Beyea

**Affiliations:** a Dalhousie University, School of Biomedical Engineering, Halifax, Nova Scotia, Canada; b Dalhousie University, Department of Diagnostic Radiology, Halifax, Nova Scotia, Canada; c Nova Scotia Health, Department of Diagnostic Imaging, Halifax, Nova Scotia, Canada; d IWK Health Centre, Halifax, Nova Scotia, Canada

**Keywords:** brain, evaluation and performance, image quality assessment, magnetic resonance imaging

## Abstract

**Purpose:**

Objective image quality metrics (IQMs) are widely used as outcome measures to assess acquisition and reconstruction strategies for diagnostic images. For nonpathological magnetic resonance (MR) images, these IQMs correlate to varying degrees with expert radiologists’ confidence scores of overall perceived diagnostic image quality. However, it is unclear whether IQMs also correlate with task-specific diagnostic image quality or expert radiologists’ confidence in performing a specific diagnostic task, which calls into question their use as surrogates for radiologist opinion.

**Approach:**

0.5 T MR images from 16 stroke patients and two healthy volunteers were retrospectively undersampled (R=1 to 7×) and reconstructed via compressed sensing. Three neuroradiologists reported the presence/absence of acute ischemic stroke (AIS) and assigned a Fazekas score describing the extent of chronic ischemic lesion burden. Neuroradiologists ranked their confidence in performing each task using a 1 to 5 Likert scale. Confidence scores were correlated with noise quality measure, the visual information fidelity criterion, the feature similarity index, root mean square error, and structural similarity (SSIM) via nonlinear regression modeling.

**Results:**

Although acceleration alters image quality, neuroradiologists remain able to report pathology. All of the IQMs tested correlated to some degree with diagnostic confidence for assessing chronic ischemic lesion burden, but none correlated with diagnostic confidence in diagnosing the presence/absence of AIS due to consistent radiologist performance regardless of image degradation.

**Conclusions:**

Accelerated images were helpful for understanding the ability of IQMs to assess task-specific diagnostic image quality in the context of chronic ischemic lesion burden, although not in the case of AIS diagnosis. These findings suggest that commonly used IQMs, such as the SSIM index, do not necessarily indicate an image’s utility when performing certain diagnostic tasks.

## 1 Introduction

The quality of an image reflects the type and degree of information it contains, and it is best measured in the context of human perception. However, subjective assessment of image quality is a time-consuming and labor-intensive process, which has led to the widespread use of objective image quality metrics (IQMs) as a surrogate for subjective scores. IQMs originated in the computer vision literature and can be thought of as mathematical formulas that objectively evaluate digital images (originally, of natural scenes) in some way that emulates human visual processing and, therefore, correlates with the image quality perceived by an average human viewer.^1^^,^^2^

In clinical applications, the humans assessing quality are radiologists, whose perceptions are based on their expertise in performing specific diagnostic tasks. The application of IQMs to the evaluation of medical images—with an assumed correlation to the perception of diagnostic quality by an expert radiologist—has become increasingly popular. Implicit in their use is the assumption that a better IQM score indicates superior quality as a medical diagnostic image.

Many factors can alter the perceived quality of an image. For example, in the context of magnetic resonance imaging (MRI), the quality of images can be affected by the presence of noise or artifacts caused by motion during the exam. MRI acquisitions can also be accelerated by reducing the amount of k-space data collected (undersampling), which often comes at the cost of reduced image quality even when using advanced reconstruction methods such as compressed sensing (CS)^3^^–^^6^ or machine learning approaches.^2^^,^^7^^–^^9^ Even if this reduction in quality is visually similar to distortions found in natural images such as photographs, the impact of these quality differences on the usability of a medical image for diagnostic purposes—e.g., as measured by the diagnostic performance or confidence of expert radiologists—remains unclear.

There are various categories of IQMs based on the information available prior to their computation, e.g., no-reference, reduced reference, and full-reference.^10^ Although no-reference and reduced reference IQMs for medical images are being developed,^11^^,^^12^ full-reference IQMs constitute the conventional approach and are computed using an available reference image and an altered image derived from the reference. Full-reference IQMs have previously been studied to understand correlations between objective and subjective quality of magnetic resonance (MR) images. This was first done using natural images with non-expert raters scoring subjectively perceived overall image quality,^13^ followed by medical MR images absent of pathology with non-expert raters.^14^ More recently, IQMs were computed on medical MR images absent of pathology with expert radiologist raters scoring overall diagnostic image quality^15^ or degradation-specific diagnostic image quality.^2^^,^^15^ The latter has also been studied using other imaging modalities, such as computed tomography (CT).^16^

Of the 10 full-reference IQMs previously investigated by Mason et al.,^15^ the strongest correlations with radiologists’ scores of overall diagnostic image quality were exhibited by the noise quality measure (NQM),^17^ the visual information fidelity (VIF) criterion,^18^ and the feature similarity (FSIM) index.^19^ These IQMs all showed better correlations than root mean square error (RMSE) and structural similarity (SSIM).^10^ This is notable given that RMSE and SSIM are two of the most common IQMs within the MRI literature, where they are used to compare different acquisition and/or reconstruction strategies, or as part of a cost-function for image quality optimization.^20^^–^^23^

However, regardless of the sensitivity of IQMs to particular types of image alterations, or their correlation with subjective human perception in general, overall image quality might not necessarily indicate an image’s usefulness for a specific diagnostic task. High objective image quality has no bearing if the image cannot serve its intended diagnostic purpose, e.g., the chosen contrast does not visualize a pathology of interest. The opposite may also be true: despite poor objective image quality due to low signal-to-noise ratio, for example, an image may be useful for a particular purpose if the contrast between key tissues remains high. A given level of noise or artifact may obscure a lesion or structure relevant to one diagnostic task yet have no bearing on the ability to visualize other structures for a different task.

This study explores several full-reference IQMs previously identified as correlating with overall diagnostic image quality^15^ and assesses how they perform when computed on images with potential clinical pathology, where expert neuroradiologists score their ability to perform diagnostic tasks as they would in a standard clinical setting. Specifically, neuroradiologists provided task-specific diagnostic confidence scores for reporting the presence or absence of acute ischemic stroke (AIS) and assigning Fazekas scores to rank the extent of chronic ischemic lesion burden.^24^ Clinical diagnosis of acute or chronic ischemic lesions is used here as an exemplar, given that spatial patterns, image contrast, and lesion conspicuity in these diagnostic cases are very different: acute ischemia typically presents in MRI as focal high contrast lesions, and chronic small vessel ischemia presents as diffuse lower contrast lesions.^25^ We hypothesize that, as in Ref. 15, the FSIM, NQM, and VIF will correlate more closely than RMSE or SSIM with neuroradiologists’ subjective scoring of diagnostic confidence, for both the acute and chronic diagnostic tasks.

This study uses MR images prospectively acquired via a head-only point-of-care 0.5 T MRI system^26^ and retrospectively accelerated. This approach was chosen because accelerated MRI is key for emergency medicine situations, such as AIS, where rapid imaging protocols are vital, and although acceleration is known to alter image quality, it may be the case that an image remains diagnostically useful for these tasks even after substantial objective alteration. Previous work on this system^27^ supported the decision to accelerate because it showed 100% congruency in scan findings for non-accelerated acute stroke imaging at 0.5 T compared with conventional MRI systems (1.5 T, 3 T). Our focus on task-specific diagnostic image quality echoes the suggestions by Washburn-Campbell et al.^28^ to both “consider the clinical questions, at hand” and assess image quality through the lens of information content as the value-add.

## 2 Material and methods

### 2.1 Data Acquisition

#### 2.1.1 Participant population, recruitment, and demographics

All participants were recruited under protocols approved by the Nova Scotia Health Research Ethics Board. Informed consent and MRI safety screening were obtained from all potential participants of this study prior to them being imaged.

Patients with clinically diagnosed AIS on initial standard-of-care CT scans were identified by an attending neurology physician during their clinical care in the emergency department (ED). These patients were imaged for this study via 0.5 T MR once they had completed their ED clinical care and were in stable condition as admitted inpatients. A total of N=12 such patients completed participation and were included in this study (5F, 7M, average age 64 years, 0.5 T MRI acquired mean/median 5.7/6 days post-clinical CT). In addition, patients with clinically suspected but unconfirmed AIS on initial standard-of-care CT scans were also recruited to the study and imaged via 0.5 T MR when they were admitted as inpatients and still in the process of receiving their clinical care but deemed stable by the treating physician. A total of N=4 such patients completed participation (3F, 1M, average age 60.5 years, 0.5 T MRI acquired mean/median 1.3/1.5 days post-clinical CT). (Of these, it was eventually determined by clinical MRI exams that two were positive for AIS and two were negative.) N=2 healthy control participants (2F, average age 27.5 years) were also recruited and imaged via 0.5 T MR.

In summary, data were acquired from a total of 18 participants (10F, 8M, average age 59 years), of which 16 were diagnosed and/or suspected AIS patients. The datasets provided to neuroradiologists therefore spanned the range of patient variation, which they may see in this clinical context: healthy (nonstroke), stroke-positive on first-line clinical CT imaging, stroke-negative on first-line clinical CT imaging, and of the latter, stroke-positive or stroke-negative on subsequent clinical MRI. This ensured that, when performing diagnostic tasks, neuroradiologists confidence scores would be representative of genuinely performing the task, rather than being based on hypotheticals, or a population known to contain solely stroke-positive images.

#### 2.1.2 Participant imaging and scan parameters

All study participants were imaged on a head-only 0.5 T MRI system (Synaptive Medical, Toronto, Ontario, Canada). Multiple sequences are included in the standard stroke protocol (identical to Ref. 27) used to image participants on this system. However, the only sequences used in this study were axial T2 fluid-attenuated inversion recovery (FLAIR) and axial diffusion-weighted imaging (DWI) with corresponding apparent diffusion coefficient (ADC) maps. This series of image datasets—T2 FLAIR and DWI/ADC—are those within the stroke imaging protocol, which are principally used clinically to diagnose AIS and chronic ischemic lesion burden. FLAIR parameters were: acquisition time 266 s; TR = 5893 ms; TE = 86 ms; TI = 1904 ms; acquired resolution 1.0  mm×1.2  mm, interpolated to 0.5  mm×0.5  mm; acquired matrix size 250×216, interpolated to 512×512; FOV=250  mm×250  mm; NEX = 3; 28 slices. DWI parameters were: acquisition time 97 s; TR = 3945 ms; TE = 83 ms; acquired resolution 2.0  mm×2.0  mm, interpolated to 0.9375  mm×0.9375  mm; acquired matrix size 120×120, interpolated to 256×256; FOV=240  mm×240  mm; b-value = $0\;s\;{\mathrm{mm}}^{-2}$ and $1000\;s\;{\mathrm{mm}}^{-2}$; 28 slices.

### 2.2 Data Processing

#### 2.2.1 Retrospective undersampling and compressed sensing reconstruction

Given that the T2 FLAIR acquisition time was almost three times longer than DWI, only the T2 FLAIR images were retrospectively accelerated. Raw, fully sampled k-space data from the prospective axial T2 FLAIR scans of all 18 participants were saved and input to a customized data pipeline in MATLAB 2021b (The Mathworks, Natick, Massachusetts, United States). This pipeline performed k-space data undersampling in the phase-encoding direction (R=1-7X, linear 2D Cartesian) and compressed sensing reconstruction (with ℓ1-wavelet regularization, λ=0.01) using the Berkeley Advanced Reconstruction Toolbox (BART).^29^ All sampling masks were generated in MATLAB using SparseMRI^30^ with parameters set such that the central eighth of k-space data were fully sampled with a polynomial decay rate of p=7. This process yielded 18 reference and 108 undersampled T2 FLAIR image datasets. Axial DWI images and the corresponding ADC maps were not accelerated and the images presented to raters were those acquired directly from the 0.5 T MRI system.

#### 2.2.2 Two-dimensional image quality metric computation

All reconstructed reference and undersampled T2 FLAIR image datasets were normalized prior to 2D IQM computations. As a result, RMSE was normalized to the intensity of the reference image, and therefore ranged from zero to one, indicating best to worst RMSE image quality scores, respectively. RMSE, SSIM, FSIM, NQM, and VIF were computed for each undersampled image slice (R=2 to 7×) based on the corresponding reference image slice (R=1×) using publicly available implementations of these IQMs in MATLAB 2021b.

### 2.3 Task-Specific Diagnostic Confidence Study with Neuroradiologist Raters

All images were de-identified and saved in the DICOM format. For neuroradiologist review, seven image datasets were generated for every participant, each consisting of a T2 FLAIR series—either the non-accelerated reference (R=1×) or one of the six undersampled image series (R=2 to 7×)—as well as the participant’s non-accelerated DWI series and its corresponding ADC map. Each dataset therefore contained all the images a radiologist would use in a clinical context for the diagnostic tasks performed.

#### 2.3.1 Neuroradiologists’ diagnostic tasks and scoring

Image dataset series were individually presented in a pseudo-random order to three fellowship-trained neuroradiologists with 4, 4, and 14 years of dedicated neuroradiology experience. To attempt to mitigate recognition bias, images were presented in batches over the course of 6 weeks, with each week’s batch curated to contain no more than two copies of the same dataset at different acceleration factors, and to ensure that a similar number of each acceleration factor was included. Radiologists were not obligated to rate all images in a batch during a single session, which may also have helped reduce the recall of particular cases.

For each series, neuroradiologists were individually asked to perform two clinically relevant diagnostic tasks specific to ischemic lesions. The acute diagnostic task was, “Using the DWI/ADC and T2 FLAIR images, would you report the presence of an acute stroke?” The chronic diagnostic task was, “What Fazekas score (0 to 3) would you report for identification of chronic ischemic lesion burden?” where 0 = absent; 1 = punctate foci, or “caps” or pencil-thin lining; 2 = beginning confluence, or smooth “halo”; and 3 = large confluent areas, or irregular periventricular signal extending into deep white matter. Neuroradiologists were also asked to rank their diagnostic confidence in performing each task on a 1 to 5 Likert scale (where 1 means 0% confidence and 5 means 100% confidence).

A total of 126 image dataset series (18 participants * 7R values) were scored by each neuroradiologist, yielding a total of 378 scores (54 total scores per R value) for each task and their corresponding diagnostic confidence. Scoring was subject to initial and recurrent calibration. Boxplots were generated for the visualization of diagnostic confidence scores.

#### 2.3.2 Data analysis

Inter-rater reliability was assessed to justify pooling diagnostic confidence scores across neuroradiologists. However, the standard metric of inter-rater reliability, Cohen’s kappa, has inherent paradoxes,^31^ which were observed in our data. Therefore, we used Gwet’s quadratic weighted agreement coefficient (AC2)^32^^,^^33^ to measure inter-rater reliability across all three neuroradiologists’ diagnostic confidence scores in performing the acute and chronic diagnostic tasks. Gwet’s AC2 accounts for the degree of disagreement by penalizing smaller disagreements to a lesser extent (e.g., Likert scores of 1 versus 5 would be penalized more heavily than Likert scores of 4 versus 5) and performs more stably than Cohen’s kappa in certain cases, such as those with known high agreement. Gwet’s AC2 values were computed in RStudio version 1.4.1106.

As neuroradiologists were in strong agreement (Gwet’s AC2 >0.80), it was deemed allowable to pool all 378 diagnostic confidence scores (3 neuroradiologists * 126 image series) for each of the acute and chronic diagnostic tasks. Gaussianity of confidence score data was determined via kurtosis computations performed in MATLAB. To assess whether diagnostic confidence drops significantly beyond a certain acceleration factor, Wilcoxon signed-rank statistical tests (performed in RStudio) were used for pairwise comparison of neuroradiologists’ pooled diagnostic confidence scores between R=1× and each R>1× for both the acute and chronic diagnostic tasks. Post-hoc Bonferroni-corrected p-values less than 0.05 were reported as significant.

### 2.4 IQM Data Analysis

#### 2.4.1 Regression analysis

Similar to previous studies,^15^^,^^16^ neuroradiologists’ diagnostic confidence scores for undersampled images were evaluated as pooled and averaged Likert scores (rescaled from 0 to 100) for both the acute and chronic diagnostic tasks. Pooling and averaging of scores were allowable by the results of the inter-rater reliability computations.

Neuroradiologists’ diagnostic confidence scores were separately plotted for each task versus objective IQM scores computed for all undersampled T2 FLAIR images. The plotted data were fit to a constrained logistic function for a nonlinear regression model in MATLAB using lsqcurvefit. The corresponding sum of squares residuals (SSR) and Spearman rank order correlation coefficient (SROCC) values were computed in MATLAB. SSR represents the logistic model’s goodness-of-fit to the data, whereas SROCC represents the correlation between the objective IQM scores and the neuroradiologists’ subjective confidence scores in performing diagnostic tasks.

For each IQM and diagnostic task, the Gaussianity of raw signed residuals between subjective scores and the nonlinear logistic regression model fit to objective IQM scores was determined via kurtosis computations performed in MATLAB.

#### 2.4.2 Statistical testing

Wilcoxon signed-rank statistical tests (performed in RStudio) were used for pairwise comparison of absolute value residuals between subjective scores and the logistic fit for each IQM for both the acute and chronic diagnostic tasks. This allows ranking of the relative performance of the IQMs in terms of their ability to capture changes in neuroradiologists’ confidence scores and thus assess task-specific diagnostic image quality. Post-hoc Bonferroni-corrected *p*-values less than 0.05 were reported as significant.

## 3 Results

**Fig. 1:**
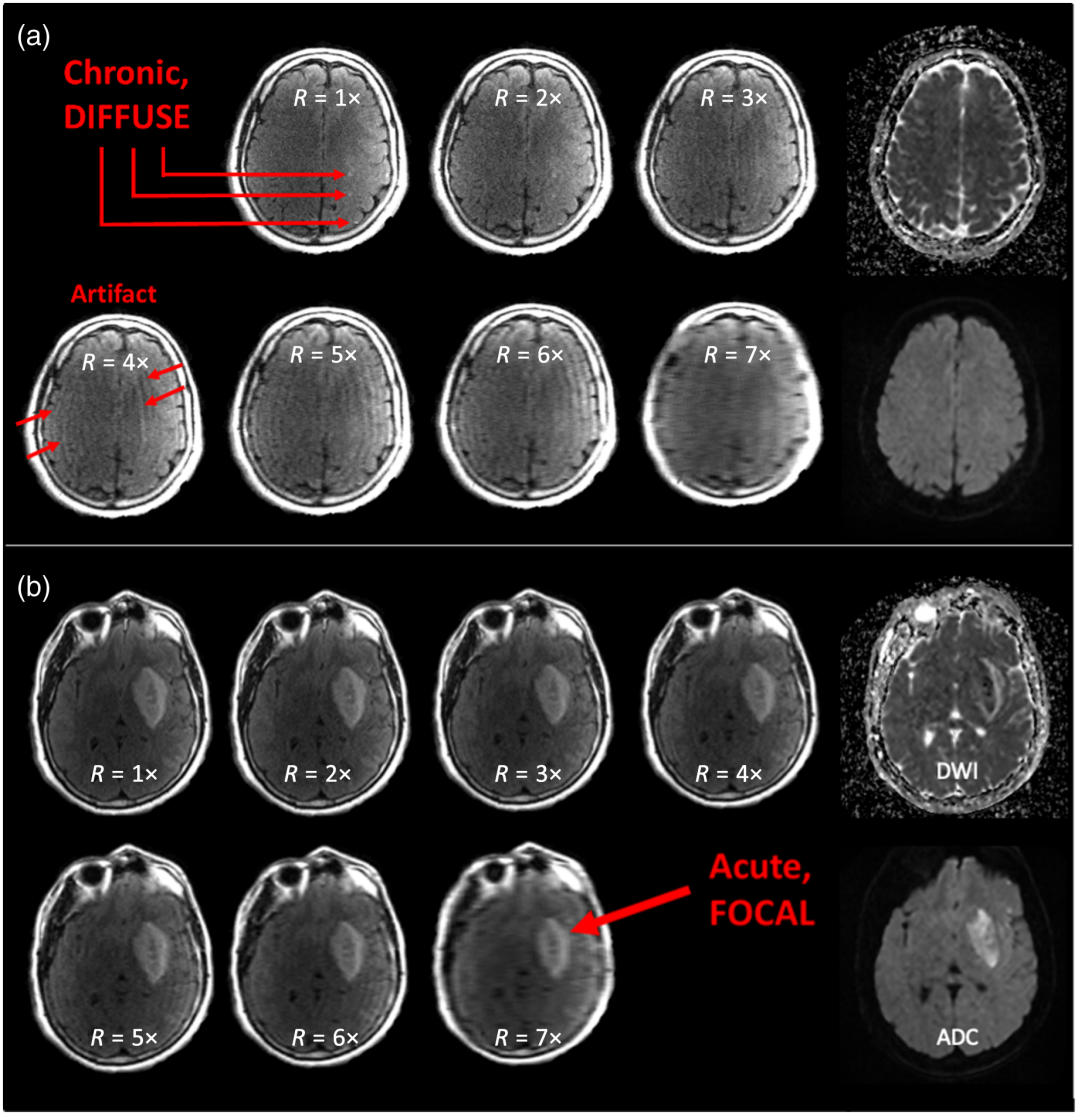
2D T2 fluid-attenuated inversion recovery (FLAIR) image slices from an accelerated MR image dataset corresponding to a representative acute ischemic stroke (AIS) patient. Unique anatomical slices show (a) chronic ischemic lesions and (b) AIS, as visualized in images with R=1× (fully sampled reference) and R=2 to 7× (undersampled images). Undersampling followed by compressed sensing reconstruction resulted in variations in image quality. Matched slices from the DWI series and ADC map are also shown for comparison.

Figure 1 shows an example of 2D slices selected from an accelerated MR image dataset corresponding to a representative AIS patient. From this dataset, two 2D slices were chosen and shown for R=1 to 7×: one at a position that visualizes the chronic ischemic lesion burden pathology [Fig. 1(a)] and one that visualizes AIS pathology [Fig. 1(b)]. These slices illustrate the impact of image acceleration via CS reconstruction on image quality and how neuroradiologists’ task-specific diagnostic confidence scores may be impacted based on the image artifacts observed. Specifically, chronic ischemic lesion burden presents with more diffuse, low-contrast image features that are more likely to be masked by CS artifacts, whereas AIS presents with more focal, high-contrast image features. Matched slices from DWI and ADC maps are also presented.

### 3.1 Neuroradiologist Raters’ Performance on Diagnostic Tasks Specific to Ischemic Lesions

For the chronic diagnostic task, Fazekas scores spanned the entire range (0 to 3), although the majority of all scores were 0 or 1. Although there is no ‘correct’ Fazekas score by which neuroradiologists’ accuracy can be assessed, the average overall raw inter-rater agreement was 51%, with only three pairwise inter-rater disagreements differing by ±2 and the rest differing by ±1. (see Figs. S1–S3 in the Supplementary Material for task scoring results.) Neuroradiologists performed the acute diagnostic task with 100% accuracy, regardless of image quality.

### 3.2 Neuroradiologist Raters’ Confidence Scoring

Figures 1(a) and 3(a) illustrate the tally of neuroradiologists’ diagnostic confidence scores in performing the chronic and acute diagnostic tasks, respectively, with each R value on a separate row. The kurtosis of neuroradiologists’ diagnostic confidence scores pooled across R=1 to 7× is 4.5 and 61.4 for the chronic and acute diagnostic tasks, respectively. Figures 1(b) and 3(b) show the corresponding boxplots of neuroradiologists’ pooled diagnostic confidence scores at each R. These panels also show the results of the Wilcoxon signed-rank test for pairwise comparison of confidence scores between R=1× and each R>1× for the (a) chronic and (b) acute diagnostic tasks. Although significant drops in neuroradiologists’ diagnostic confidence are observed beyond R=3× for the chronic diagnostic task, no significant changes were observed up to R=7× for the acute diagnostic task.

**Fig. 2:**
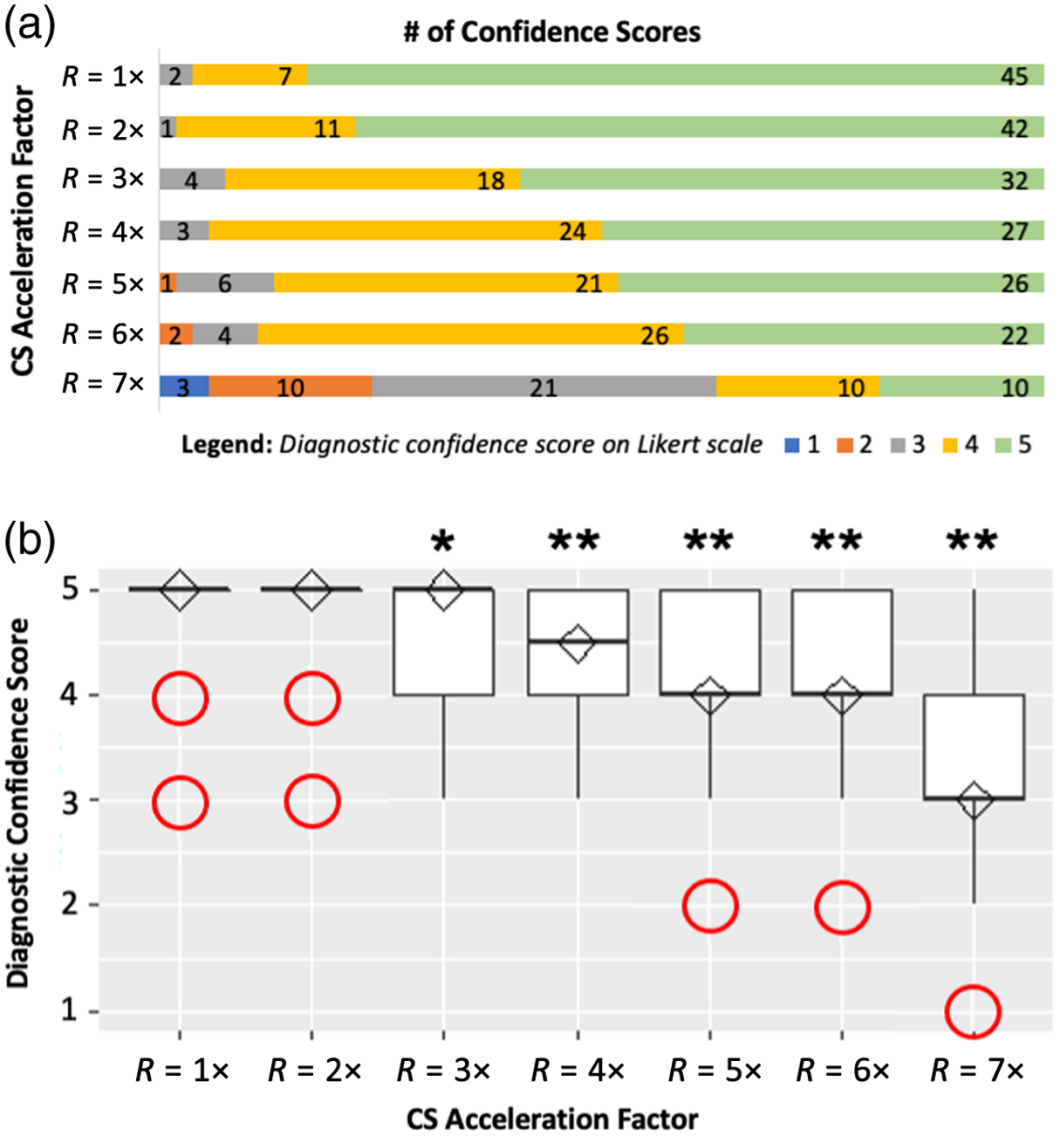
(a) Neuroradiologist raters’ diagnostic confidence scores in assigning Fazekas scores to rank the extent of chronic ischemic lesion burden (i.e., chronic diagnostic task) on a five-point Likert scale: 1 = 0% confident, 2 = 25% confident, 3 = 50% confident, 4 = 75% confident, 5 = 100% confident. (b) Neuroradiologist raters’ pooled diagnostic confidence scores for the chronic diagnostic task, plotted versus R, and represented as boxplots. Diamonds represent the median, and circles are diagnostic confidence score outliers. Asterisks indicate acceleration factors where confidence scores differ significantly from R=1×, with * indicating p<0.05 and ** indicating p<0.001. CS, compressed sensing.

**Fig. 3:**
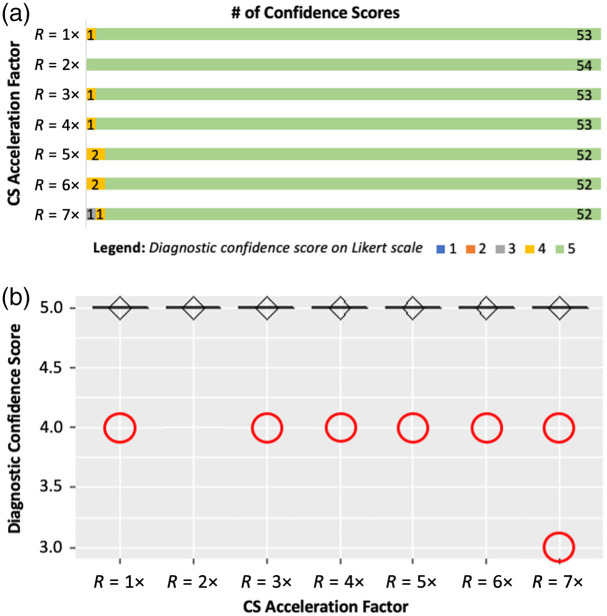
(a) Neuroradiologist raters’ diagnostic confidence scores in reporting the presence or absence of AIS (i.e., acute diagnostic task) on a five-point Likert scale: 1 = 0% confident, 2 = 25% confident, 3 = 50% confident, 4 = 75% confident, 5 = 100% confident. (b) Neuroradiologist raters’ pooled diagnostic confidence scores for the acute diagnostic task, plotted versus R, and represented as boxplots. Diamonds represent the median, and circles are diagnostic confidence score outliers. None of the scores for R>1× were significantly different than those for R=1× (p>0.9). CS, compressed sensing.

Agreement between neuroradiologists based on pairwise Gwet’s AC2, along with 95% confidence intervals, is provided in Table 1. All values for the chronic diagnostic task [Table 1 (a)] indicate “almost perfect” rater agreement, whereas all values for the acute diagnostic task [Table 1 (b)] indicate “almost perfect” or “perfect” rater agreement. (Inter-rater agreement for the chronic task does change as a function of acceleration factor; see, Fig. S4 in the Supplementary Material.)

**Table 1:** Pairwise inter-rater reliability between each of the three neuroradiologist raters’ diagnostic confidence scores in reporting (a) Fazekas scores for chronic ischemic lesion burden and (b) presence/absence of AIS, via Gwet’s AC2 ± 95% confidence interval. AC2: quadratic weighted agreement coefficient.

	(a) Chronic diagnostic task				(b) Acute diagnostic task		
	Rater 1	Rater 2	Rater 3		Rater 1	Rater 2	Rater 3
**Rater 1**	—	0.927 ± 0.031	0.853 ± 0.050	**Rater 1**	—	1.00 ± 0.00	0.994 ± 0.006
**Rater 2**	0.927 ± 0.031	—	0.839 ± 0.048	**Rater 2**	1.00 ± 0.00	—	0.994 ± 0.006
**Rater 3**	0.853 ± 0.050	0.839 ± 0.048	—	**Rater 3**	0.994 ± 0.006	0.994 ± 0.006	—

### 3.3 Regression Analysis

**Fig. 4:**
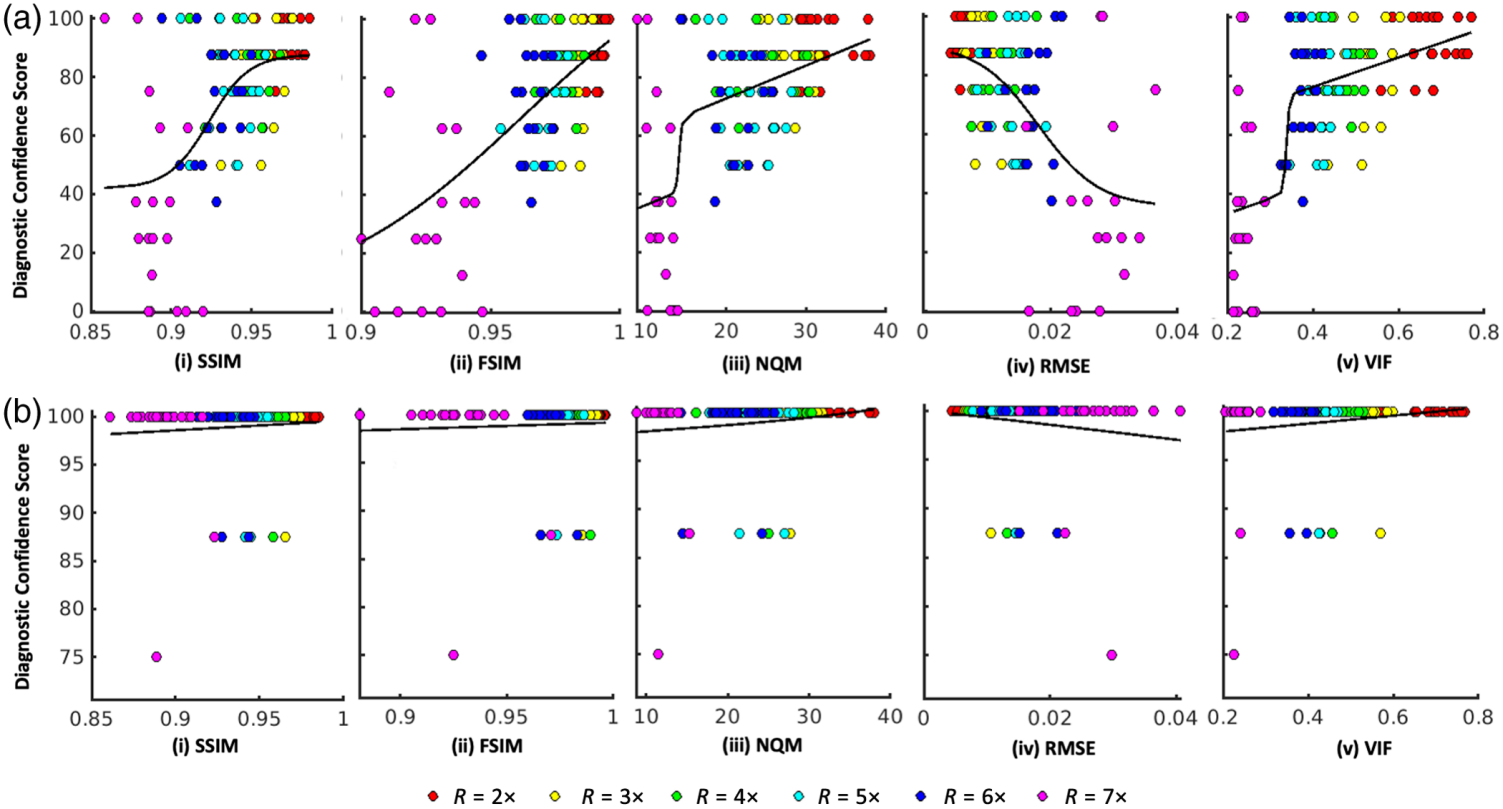
Neuroradiologist raters’ diagnostic confidence scores corresponding to the (a) chronic and (b) acute diagnostic tasks, both versus image quality metric (IQM) scores computed for all undersampled T2 fluid-attenuated inversion recovery (FLAIR) images. Plotted data were fit to a constrained logistic function for a nonlinear regression model. The y-axis shows confidence scores rescaled from 1 to 5 Likert scale values to 0 to 100. Marker colors denote scores from images reconstructed with different acceleration factors from R=1× to R=7×.

Figure 4 plots neuroradiologists’ diagnostic confidence scores corresponding to the (a) chronic and (b) acute diagnostic tasks, versus IQM scores computed for all undersampled T2 FLAIR images and fit to a constrained logistic function for a nonlinear regression model. Note that a higher SSIM, FSIM, NQM, and VIF score indicates higher objective image quality, whereas an RMSE score of 0 corresponds to the highest objective image quality.

The results for the corresponding SSR values, kurtosis values of the raw signed residuals, and SROCC values are shown in Table 2 for the (a) chronic and (b) acute diagnostic tasks. If the kurtosis value of the raw signed residuals was between 2.0 to 4.0 (inclusive), the distribution of the residuals was deemed to be Gaussian. (To demonstrate the distribution of the raw signed residuals, see Figs. S5–S6 in the Supplementary Material, which show the histograms associated with each IQM and diagnostic task.)

**Table 2:** Performance of nonlinear regression model fits to objective IQM scores. (i) Sum of squared residuals (SSR), with lower values indicating a better model fit. (ii) Kurtosis of the raw signed residuals indicates whether residuals are Gaussian (2 to 4) or non-Gaussian (>4). (iii) Spearman rank order correlation coefficient (SROCC) between subjective and objective data, with SROCC = ±1 indicating perfect correlation, and SROCC = 0 indicating no correlation.

	(a) Chronic diagnostic task					(b) Acute diagnostic task				
	SSIM	FSIM	NQM	RMSE	VIF	SSIM	FSIM	NQM	RMSE	VIF
**(i) SSR**	$4.78\times 10^{4}$	$4.12\times 10^{4}$	$4.28\times 10^{4}$	$4.74\times 10^{4}$	$3.58\times 10^{4}$	1.13	1.13	1.12	1.10	1.11
**(ii) Kurtosis**	4.3	4.2	3.9	4.2	4.8	18.5	18.7	17.5	17.4	17.5
**(iii) SROCC**	0.50	0.53	0.46	−0.48	0.55	0.05	0.01	0.10	−0.17	0.12

### 3.4 Statistical Testing

The relative performance of the IQMs in terms of their ability to capture changes in neuroradiologists’ confidence scores, and thus assess task-specific diagnostic image quality, is reflected in Tables 3 and 4 for the chronic and acute diagnostic tasks, respectively. These tables show the results of the Wilcoxon signed-rank test for pairwise comparison of absolute value residuals between subjective scores and the logistic fits for the corresponding IQMs. Significant p-values (p<0.05) indicate that the IQM (y) in a given row of the table performs significantly better than the IQM (x) in the column (see Figs. S7–S9 in the Supplementary Material for illustrative examples of the three respective cases). In the chronic diagnostic task, VIF performed statistically better than both NQM and RMSE, with all other pairs of IQMs showing equivalent relative performance (no significant differences). Due to consistent radiologist confidence at all degradations tested, no IQMs showed significant differences in performance for the acute diagnostic task.

**Table 3:** Wilcoxon signed-rank statistical test results for pairwise comparison of IQM performance, based on absolute value residuals between subjective scores for the chronic diagnostic task, and the logistic fits for the corresponding IQMs. p-Values are Bonferroni corrected for multiple comparisons. Significant p-values (<0.05, denoted by boldface type) indicate that the IQM (y) in the row outperforms the IQM in the column (x).

		(x)				
		SSIM	FSIM	NQM	RMSE	VIF
(y)	SSIM	—	1.000	1.000	0.1785	1.000
	FSIM	1.000	—	0.2017	1.000	1.000
	NQM	1.000	1.000	—	0.7728	1.000
	RMSE	0.2402	0.3084	1.000	—	1.000
	VIF	1.000	1.000	**0.0042**	**0.0172**	—

**Table 4:** Wilcoxon signed-rank statistical test results for pairwise comparison of IQM performance, based on absolute value residuals between subjective scores for the acute diagnostic task, and the logistic fits for the corresponding IQMs. p-Values are Bonferroni corrected for multiple comparisons.

		(x)				
		SSIM	FSIM	NQM	RMSE	VIF
(y)	SSIM	—	1.000	1.000	1.000	1.000
	FSIM	0.7904	—	1.000	1.000	1.000
	NQM	0.4688	1.000	—	1.000	1.000
	RMSE	0.3156	0.7232	1.000	—	1.000
	VIF	1.000	0.3490	1.000	1.000	—

## 4 Discussion

Objective IQMs that were initially developed for natural images have increasingly been applied in the context of medical imaging. Correlating IQMs with radiologists’ confidence scores is important to develop efficient study protocols and novel reconstruction algorithms without requiring lengthy questionnaire sessions with radiologists, thereby reducing the need for their valuable and limited time. Previous studies have sought to transition from generic distortions and non-expert raters,^14^^,^^16^ toward realistic degradations in quality and trained radiologist evaluators.^2^^,^^15^

This study takes two further steps from previous work to increase the clinical relevance of IQMs. First, image quality degradations are not applied only to images absent of pathology, but to image datasets that include ED patients with AIS and/or chronic ischemic lesion burden. Second, instead of assessing overall diagnostic image quality, the inclusion of pathology in this study allows expert radiologists to assess the task-specific diagnostic quality of images used to perform standard clinical tasks related to reporting/identification of acute and chronic ischemic lesions. These choices are more realistic to practical application and better approximate the impact of prospective acceleration in a clinical setting.

We chose to study a subset of IQMs that are known to correlate with radiologists’ scores of overall diagnostic image quality (FSIM, NQM, VIF),^15^ as well as those IQMs most commonly used in MRI literature (RMSE, SSIM).^22^^,^^34^ These certainly do not represent the full range of IQMs available^2^ nor even the “best available” in terms of performance. The main objective of this study was not to rank IQM performance but to conduct an initial examination of the extent to which IQMs correlate with radiologist confidence in performing a diagnostic task and clarify the ability of IQMs to assess task-specific diagnostic image quality. Future work will continue to explore the degree to which emerging IQMs correlate with radiologist performance, including IQMs based on deep learning models such as DISTS^35^^,^^36^ or no-reference IQMs.^11^

For both diagnostic tasks, the residuals of the model fit for each IQM are predominantly non-Gaussian, as evidenced by the kurtosis values in Table 2, row (ii). For the acute diagnostic task, the kurtosis values for the raw signed residuals were much greater than 4.0 regardless of IQM, meaning that none of them represent a normal distribution. This necessitated the use of the Wilcoxon signed-rank tests to evaluate the relative performance of each IQM. These tests were also used for the chronic diagnostic task as four of the five IQMs had kurtosis values greater than 4.0 for the distribution of their raw signed residuals, whereas the last had a kurtosis of 3.9, indicating a distribution very close to non-Gaussian.

When assessing the quality of MR images used to perform the chronic diagnostic task, it was observed that the conspicuity of chronic ischemic lesions on the T2 FLAIR images was not maintained, especially at acceleration beyond R=3×, due to low contrast between key tissues representing this diffuse ischemic pathology (Fig. 1). As seen in Fig. 2, neuroradiologists’ diagnostic confidence scores are distributed across all Likert scores, with a trend towards lower scores as R increases. Based on the Wilcoxon signed-rank test, although there was no significant change in neuroradiologists’ diagnostic confidence in performing the chronic diagnostic task from R=1× to 2× (p=1.00), there were significant changes from R=3 to 7× (all p-values <0.02), as more severe undersampling artifacts begin to mask the chronic, diffuse ischemic lesions.

Accordingly, the correlations between IQMs and neuroradiologist ratings are relatively strong (Fig. 4), and it is possible to order the IQMs in terms of performance (Table 2). From best to worst, the rank orders are as follows: (1) VIF, (2) FSIM, (3) NQM, (4) RMSE, and (5) SSIM, based on the SSR values; and (1) VIF, (2) FSIM, (3) SSIM, (4) RMSE, and (5) NQM based on the SROCC values. In particular, the VIF and FSIM indices, which performed well in terms of overall diagnostic image quality in Ref. 15 also performed strongly in this diagnostic task. However, none of the IQMs in this study were significantly better than any other for the chronic diagnostic task (all p-values >0.18), except for VIF, which performed better than RMSE (p=0.017) and NQM (p=0.004).

By contrast, the radiologist’s performance of the acute diagnostic task was essentially unaffected by the acceleration of the T2 FLAIR image. Neuroradiologists’ diagnostic confidence scores for the acute task (Fig. 3) are mainly Likert scores of 5, with a minimal distribution of scores 3 to 4, and no scores below 3. With the Wilcoxon signed-rank test, there was no significant change (all p-values >0.95) in neuroradiologists’ diagnostic confidence in performing the acute diagnostic task, even as images were undersampled up to R=7×. However, as the acceleration factor increases, the resulting T2 FLAIR image is increasingly prone to resolution loss and noise-like aliasing artifacts (Fig. 1), and IQM scores change accordingly. As a result, none of the IQMs showed a strong correlation with neuroradiologists’ diagnostic confidence (Fig. 4).

A key limitation of this study is that the impact of acceleration on the DWI image (and on the corresponding ADC map) was not explored. The T2 FLAIR acquisition was the one chosen to accelerate due to its longer acquisition time of 266 s; as the DWI acquisition was already only 97 s, its acceleration was deemed less impactful for the purpose of this study. Nonetheless, based on recommendations in the literature^24^^,^^37^ and input from clinicians, it was decided to present radiologists with the T2 FLAIR and DWI/ADC images because these are the sequences used to identify acute and chronic ischemic lesions in clinical practice. Although clinicians refer to all available images, especially in challenging cases, the high contrast representing focal ischemic pathology on the DWI/ADC map likely contributes to the robustness of radiologists’ confidence scores despite increasing degradation of the T2 FLAIR image. This highlights the need to use IQMs with knowledge of what diagnostic tasks a particular image might be used for; although high acceleration of an image may be permissible in one context, it may be detrimental in another. An IQM, which is to become a useful surrogate for radiologist opinion, must not only capture the salient features of an image but also be applied to the image(s) that best present those features to a radiologist, for a particular diagnostic task.

This study has several other limitations, which should be considered when attempting to expand upon it or reproduce its approach in different clinical contexts. The small sample size increases the likelihood that radiologists will recall a particular case when reviewing images degraded in multiple ways, despite steps taken to mitigate recognition bias as described above. A larger pool of images increases the number of truly independent measurements and would improve statistical comparison of IQM performance, whereas a larger number of raters would allow for modeling of the impact of radiologist experience or subspecialty. A full evaluation of the appropriateness of a given IQM for a particular diagnostic task must also be robust to changes in parameters, MRI scanner manufacturer, and other factors that vary across sites.

Acknowledging that there are many ways to undersample and reconstruct images,^38^^–^^40^ the rationale behind choosing the particular reconstruction method employed herein was one of feasibility, not optimality. CS is one example of a well-known and tested implementation of MR image acceleration and reconstruction, and the particulars of the reconstruction options are less relevant than the assessment of neuroradiologists’ task-specific diagnostic confidence versus IQMs—the latter of which maintains its importance regardless of which specific acceleration and reconstruction choices are made.

Similarly, although many IQM studies^14^^–^^16^ model correlations between objective and subjective scores with a logistic function, this may not be the preferred model in every situation, especially those with almost degenerate distributions, as in the acute diagnostic task. As the relationship between objective and subjective scores depends on the diagnostic task, if this work leads to clinically translatable studies with specific anatomies, pathologies, and diagnostic tasks, the models themselves may need to become more nuanced. Future studies should not be constrained by a logistic fit model but, instead, by the diagnostic task they wish to examine.

Understanding that all of these choices affect relative IQM performance, the key in future work is to be specific to the diagnostic task rather than pursuing a catch-all optimization. Although a logical progression of this work includes using the logistic fit results to assess the streamlining of images shown to radiologists, other models should be assessed in future work. For example, the implementation of AI/machine learning models, such as those applied in Ref. 2, may be beneficial to address the case-by-case nature of correlating objective IQM scores with specific diagnostic tasks. We continue to investigate how IQMs behave with respect to other image alterations, including those prospectively derived in clinically practical settings, to provide more insight into the operations of these metrics.

## 5 Conclusions

Above an acceleration factor of R=3×, significant decreases in neuroradiologists’ diagnostic confidence were observed for the diagnostic task of assigning Fazekas scores, which correlated with a drop in objective image quality. In terms of modeling neuroradiologists’ diagnostic confidence, VIF performed statistically better than both RMSE and NQM for the chronic diagnostic task. In comparison, for the acute diagnostic task of reporting the presence or absence of AIS, neuroradiologist performance was unchanged even as T2 FLAIR images were retrospectively accelerated up to R=7×. As a result, despite a decrease in objective image quality measured by the selection of IQMs tested, none of the IQMs strongly correlated with neuroradiologists’ diagnostic confidence for the acute diagnostic task.

These conclusions regarding IQM performance only apply to the specific case in this study, i.e., the given CS reconstruction at the range of acceleration factors implemented on the particular T2 FLAIR sequence and the chosen types of ratings based on the datasets provided. The relative performance of these IQMs is likely not generalizable to other diagnostic tasks.

More generally, this study concludes that all IQMs must be evaluated in a task-specific context, and it cannot be assumed that an IQM will perform well for one anatomical area or clinical question simply because it performed well for another. Critically, studies using SSIM, for example, as a surrogate for the diagnostic utility of a new imaging technique or reconstruction method, cannot assume that SSIM will always correlate with what a radiologist observer would perceive concerning an image’s diagnostic quality and, most importantly, with their confidence in using that image to perform a clinical task.

The fact that none of the IQMs tested—RMSE, SSIM, VIF, FSIM, or NQM—correlated with neuroradiologists’ diagnostic confidence for AIS, but all correlated to varying degrees with diagnostic confidence for chronic ischemic lesion burden, shows that IQM performance does not necessarily indicate an image’s usefulness for a specific diagnostic task. This finding may inform future studies that develop novel objective IQMs that can act as a reliable surrogate measure for radiologists’ opinions, which is key to designing optimal medical imaging protocols that retain high diagnostic capability without requiring extensive reliance on radiologist raters.

## Supplementary Material

10.1117/1.JMI.12.5.051803.s01

## Data Availability

The data that support the findings of this article are not publicly available; the REB-approved protocols under which the data were collected do not allow the distribution of medical images collected for research purposes. Although the code used to analyze the data is not currently in a publicly available repository, it can be requested from the corresponding author.
