# Supplementary material for: Correlation of objective image quality metrics with radiologists’ diagnostic confidence depends on the clinical task performed
Source: J Med Imaging (Bellingham). 2025 Apr 11;12(5):051803. doi: 10.1117/1.JMI.12.5.051803 (PMC11991859; doi:10.1117/1.JMI.12.5.051803)
Supplement: Supplementary file 1 [file JMI_012_051803_SD001.docx]

SUPPLEMENTARY MATERIAL
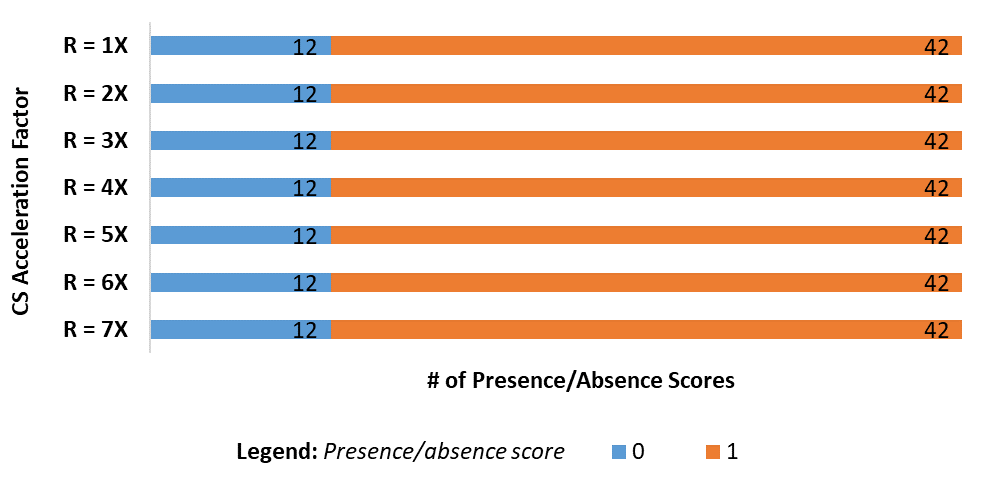


Fig. S.1. Blinded neuroradiologist raters' binary scores in reporting the presence (= 1) or absence (= 0) of acute ischemic stroke (AIS). The 3 raters each scored 18 participant image dataset series at each of the 7 acceleration factors (R). Since the acute diagnostic task involved binary decision-making, raters were either accurate or inaccurate in their performance. All raters achieved 100 % accuracy for 378 total scores in performing the acute diagnostic task regardless of image quality across R = 1-7X, and thus the average raw inter-rater agreement across all neuroradiologists for 126 image dataset series was 100 %.


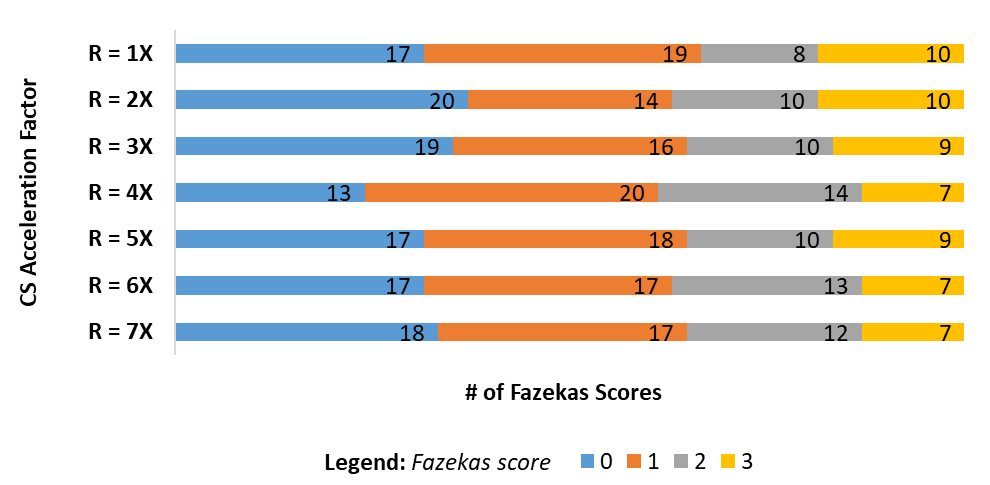


Fig. S.2. Blinded neuroradiologist raters’ Fazekas scores (i.e., reporting identification of chronic ischemic lesion burden) on the following Fazekas scale: 0 = absent; 1 = punctate foci, or “caps” or pencil-thin lining; 2 = beginning confluence, or smooth “halo”; 3 = large confluent areas, or irregular periventricular signal extending into deep white matter. The 3 raters each scored 18 participant image dataset series at each of the 7 acceleration factors (R). Since the chronic diagnostic task involved the inherently subjective Fazekas scale, there is no gold standard for judging accuracy and thus neuroradiologists’ accuracy in performing the chronic diagnostic task could not be calculated. Across R = 1-7X image quality, raters’ raw Fazekas scores span the entire range (0-3), with the majority of all scores being 0 or 1.


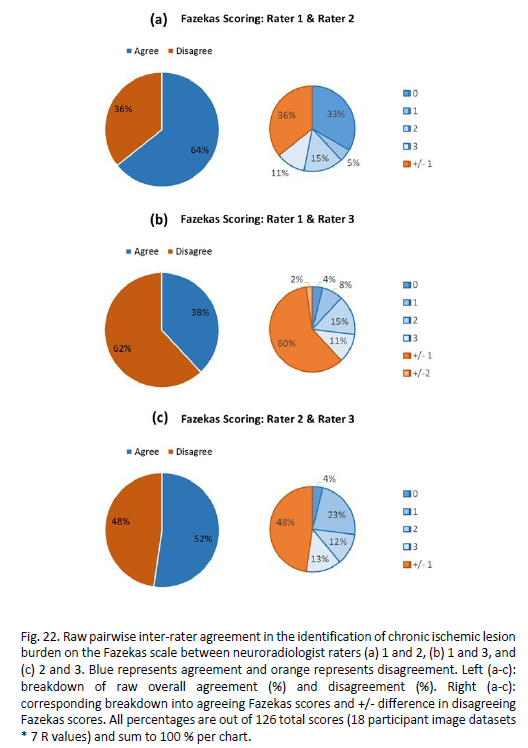


Fig. S.3. Raw pairwise inter-rater agreement in the identification of chronic ischemic lesion burden on the Fazekas scale between neuroradiologist raters (a) 1 and 2, (b) 1 and 3, and (c) 2 and 3. Blue represents agreement and orange represents disagreement. Left (a-c): breakdown of raw overall agreement (%) and disagreement (%). Right (a-c): corresponding breakdown into agreeing Fazekas scores and +/- difference in disagreeing Fazekas scores. All percentages are out of 126 total scores (18 participant image dataset series * 7 acceleration factors) and sum to 100 % per chart. Average raw inter-rater agreement across all raters is 51 %. All pairwise inter-rater disagreements are only different by +/- 1 Fazekas score, except for (b) where 3 scores were different by +/- 2.

Fig. S.4. Changes in inter-rater agreement of diagnostic confidence scores for the chronic lesion burden diagnostic task, as a function of acceleration factor (R). Agreement is measured by Gwet’s AC2, with the reported value being the smallest of the three pairwise agreements between radiologist raters. Error bars are based on the 95% confidence interval. None of the differences are statistically significant due to the small sample size after the data is separated by acceleration factor.


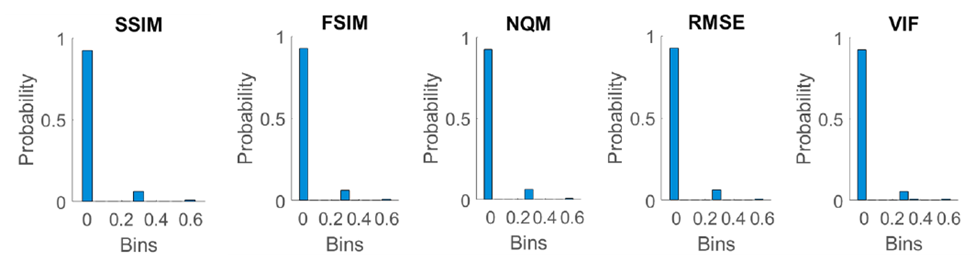


Fig. S.5. Histogram plots of the raw signed residuals associated with the non-linear regression model fit to the objective IQM scores with respect to neuroradiologist raters’ subjective diagnostic confidence scores in performing the acute diagnostic task. If the kurtosis value of the raw signed residuals was between 2.0 to 4.0 (inclusive) then the distribution of the residuals was deemed to be Gaussian. Corresponding to kurtosis values much greater than 4.0, none of the plots represent a normal distribution.

**
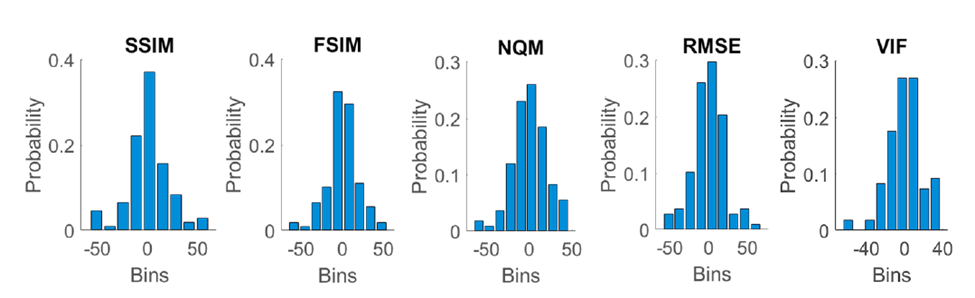
**

Fig. S.6. Histogram plots of the raw signed residuals associated with the non-linear regression model fit to the objective IQM scores with respect to neuroradiologist raters’ subjective diagnostic confidence scores in performing the chronic diagnostic task. If the kurtosis value of the raw signed residuals was between 2.0 to 4.0 (inclusive) then the distribution of the residuals was deemed to be Gaussian. Corresponding to kurtosis values only slightly greater than 4.0, the SSIM, FSIM, RMSE, and VIF plots, although close to Gaussian, technically represent a non-Gaussian distribution. With a kurtosis value of 3.9, the NQM plot represents a Gaussian distribution.


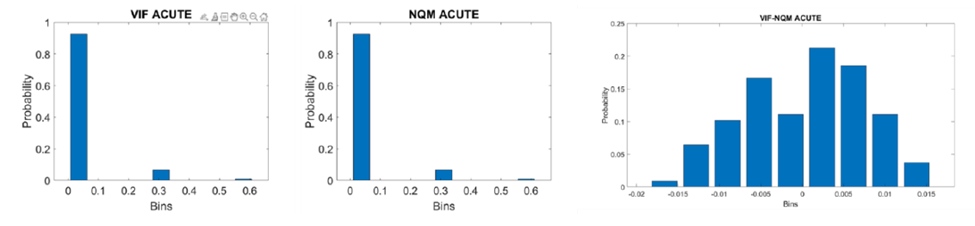


Fig. S.7. Histogram plots (left to right), corresponding to the acute diagnostic task: VIF absolute residuals (*= x*); NQM absolute residuals (*= y*); and distribution of difference between absolute residuals for VIF and NQM (*= x – y*). This illustrates *Case 1: x – y symmetric about 0* of the Wilcoxon signed-rank statistical test, where the median of the difference between x and y pairwise comparisons is symmetric about zero (*= 0*). The plots illustrate *= 0*, so according to the Wilcoxon signed-rank test, VIF neither performs better nor worse than NQM for the acute diagnostic task.


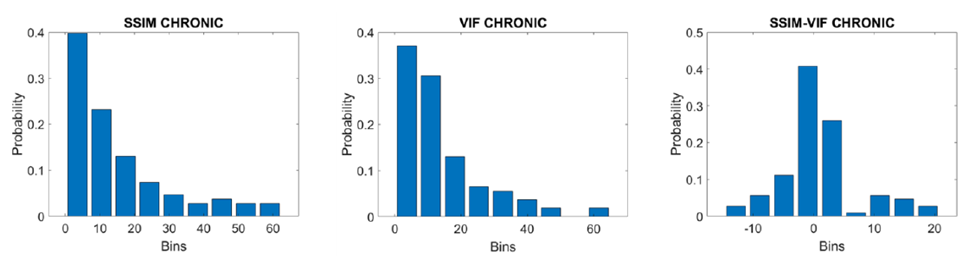


Fig. S.8. Histogram plots (left to right), corresponding to the chronic diagnostic task: SSIM absolute residuals (*= x*); VIF absolute residuals (*= y*); and distribution of difference between absolute residuals for SSIM and VIF (*= x – y*). This illustrates *Case 2: x – y > 0* of the Wilcoxon signed-rank statistical test, where the median of the difference between x and y pairwise comparisons is statistically significantly greater than zero (*> 0*). The plots illustrate *> 0*, so according to the Wilcoxon signed-rank test, SSIM performs worse than VIF for the chronic diagnostic task.


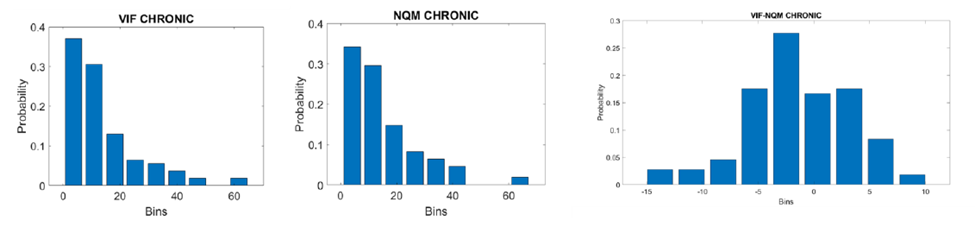


Fig. S.9. Histogram plots (left to right), corresponding to the chronic diagnostic task: VIF absolute residuals (*= x*); NQM absolute residuals (*= y*); and distribution of difference between absolute residuals for VIF and NQM (*= x – y*). This illustrates *Case 3: x – y < 0* of the Wilcoxon signed-rank statistical test, where the median of the difference between x and y pairwise comparisons is statistically significantly less than zero (*< 0*). The plots illustrate *< 0*, so according to the Wilcoxon signed-rank test, VIF performs better than NQM for the chronic diagnostic task.
